# Dynamics-Derived Insights into Complex Formation between the CXCL8 Monomer and CXCR1 N-Terminal Domain: An NMR Study

**DOI:** 10.3390/molecules23112825

**Published:** 2018-10-31

**Authors:** Prem Raj B. Joseph, Leo Spyracopoulos, Krishna Rajarathnam

**Affiliations:** 1 Department of Biochemistry and Molecular Biology, The University of Texas Medical Branch, Galveston, TX 77555, USA; 2 Sealy Center for Structural Biology and Molecular Biophysics, The University of Texas Medical Branch, Galveston, TX 77555, USA; 3 Department of Biochemistry, University of Alberta, Edmonton, AB T6G 2H7, Canada; ls5@ualberta.ca

**Keywords:** nuclear magnetic resonance (NMR), dynamics, ^15^N-relaxation, chemokine, CXCL8, CXCR1, G protein-coupled receptor (GPCR), receptor interactions, isothermal titration calorimetry (ITC)

## Abstract

Interleukin-8 (CXCL8), a potent neutrophil-activating chemokine, exerts its function by activating the CXCR1 receptor that belongs to class A G protein-coupled receptors (GPCRs). Receptor activation involves interactions between the CXCL8 N-terminal loop and CXCR1 N-terminal domain (N-domain) residues (Site-I) and between the CXCL8 N-terminal and CXCR1 extracellular/transmembrane residues (Site-II). CXCL8 exists in equilibrium between monomers and dimers, and it is known that the monomer binds CXCR1 with much higher affinity and that Site-I interactions are largely responsible for the differences in monomer vs. dimer affinity. Here, using backbone ^15^N-relaxation nuclear magnetic resonance (NMR) data, we characterized the dynamic properties of the CXCL8 monomer and the CXCR1 N-domain in the free and bound states. The main chain of CXCL8 appears largely rigid on the picosecond time scale as evident from high order parameters (*S*^2^). However, on average, *S*^2^ are higher in the bound state. Interestingly, several residues show millisecond-microsecond (ms-μs) dynamics only in the bound state. The CXCR1 N-domain is unstructured in the free state but structured with significant dynamics in the bound state. Isothermal titration calorimetry (ITC) data indicate that both enthalpic and entropic factors contribute to affinity, suggesting that increased slow dynamics in the bound state contribute to affinity. In sum, our data indicate a critical and complex role for dynamics in driving CXCL8 monomer-CXCR1 Site-I interactions.

## 1. Introduction

Chemokines constitute the largest subfamily of cytokines that play diverse and fundamental roles from trafficking immune cells including neutrophils and organogenesis to combating infection and injury [1,2]. On the basis of conserved cysteines, chemokines are broadly classified as either CXC or CC. CXCL8 belongs to a subset of CXC chemokines that are characterized by the conserved N-terminal ‘ELR’ motif. The ELR chemokines play important roles in recruiting neutrophils in response to infection and injury. They exist in equilibrium between monomers and dimers, and function as ligands for CXCR1 and CXCR2 receptors that belong to class A G protein-coupled receptors (GPCRs) [3,4,5,6]. Receptor activation involves two distinct interactions—between the chemokine N-terminal loop (N-loop) and receptor N-terminal residues (Site-I), and between the chemokine N-terminal ELR and receptor extracellular residues (Site-II) [7,8,9].

**Figure 1:**
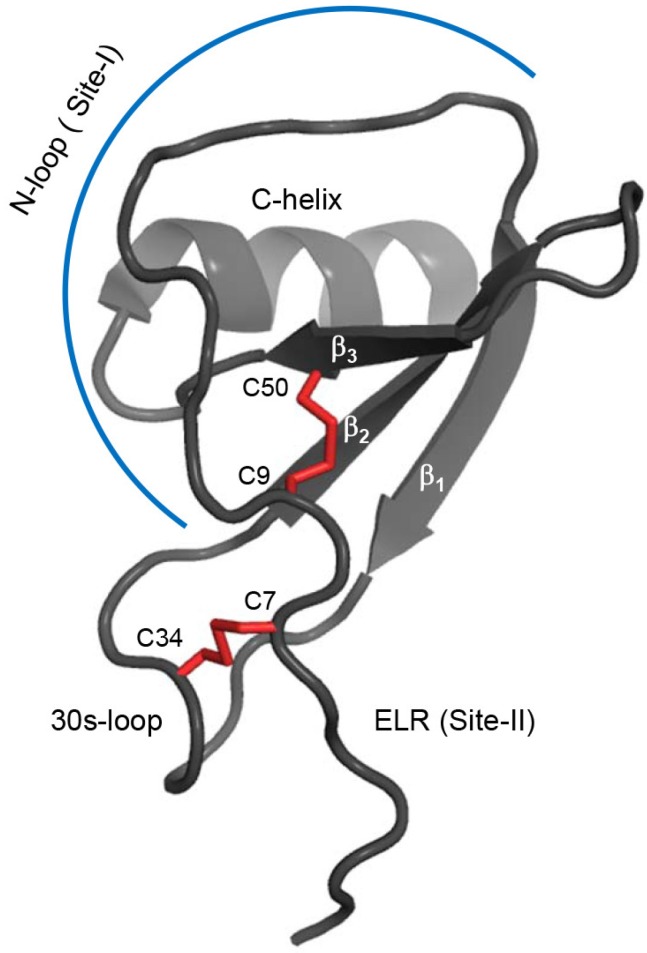
A schematic of the CXCL8 monomer (PDB ID: 5WDZ) showing the different secondary structural elements and the receptor binding sites (Site-1 and Site-II). The disulfide bonds are shown as red sticks.

Studies using CXCL8 variants that are trapped either in the monomeric or dimeric state have shown that both forms bind the CXCR1 receptor, but that the monomer alone functions as the high affinity ligand [4,10]. The structure of the CXCL8 monomer is similar to that of the monomer in the dimer (Figure 1), except the C-terminal helical residues that are structured in the dimer are unstructured in the monomer [11,12,13]. CXCL8 structures reveal that the N-loop is structured but conformationally flexible (Figure 1). The CXCR1 structure indicates that the N-terminal domain (N-domain) is unstructured and highly dynamic [14,15]. Considering the CXCL8 and CXCR1 residues that mediate Site-I interactions are unstructured and/or conformationally flexible, residue-specific descriptions of the time scales for the underlying motions would provide critical insights for understanding the molecular basis underlying binding and affinity.

Solution nuclear magnetic resonance (NMR) spectroscopy has proven to be quite powerful for providing atomistic description of motions from picosecond to millisecond time scales [16,17,18,19]. The NMR relaxation measurements of the wild type (WT) CXCL8 dimer have also shown that the N-loop residues are conformationally flexible [20,21]. However, a description of residue-specific dynamics for an intact GPCR using solution NMR methods is beyond reach at this time due to experimental limitations. Alternate strategies such as a ‘divide and conquer’ approach using the CXCR1 N-domain peptide for describing Site-I interactions have proven to be quite useful [22,23,24,25,26,27,28,29,30,31]. Binding interactions of the CXCL8 monomer to a soluble CXCR1 N-domain peptide and to a longer CXCR1 peptide that includes the first transmembrane (TM1) residues are essentially the same, indicating that peptides corresponding to the extracellular domain are sufficient for characterizing Site-I interactions [22]. Studies using the CXCR1 N-domain peptide have also shown that Site-I interactions play an important role in determining monomer vs. dimer affinity [25,26].

In this study, we characterized the backbone dynamics of a designed CXCL8 monomer and of the CXCR1 N-domain in the free and the bound states by measuring ^15^N-longitudinal (*R*_1_) and ^15^N-transverse (*R*_2_) relaxation rates, and the heteronuclear {^1^H}-^15^N nuclear Overhauser effect (NOE) [32]. We also characterized the millisecond-microsecond (ms-μs) backbone dynamics from ^15^N Carr–Purcell–Meiboom–Gill (CPMG) relaxation dispersion NMR experiments [33,34]. Furthermore, the kinetics of binding between the CXCL8 monomer and CXCR1 N-domain were determined through NMR-monitored titration with subsequent line shape analyses using the Bloch–McConnel equations for two-site chemical exchange [35,36]. Our data indicate that both nanosecond-picosecond (ns–ps) and millisecond-microsecond (ms–μs) time scale dynamics mediate Site-I interactions. Most CXCL8 monomer residues are highly structured in the free and bound states as is evident from high order parameters (*S*^2^), with *S*^2^ on an average being higher in the bound state. Interestingly, several residues in the binding domain and within its proximity show slow ms-μs dynamics only in the bound state. The CXCR1 N-domain is unstructured in the free state but structured in the bound state with a range of dynamics. We also characterized the thermodynamics of binding using isothermal titration calorimetry (ITC). The ITC data show that both enthalpic and entropic factors contribute to binding affinity, suggesting fast and slow dynamics play opposing roles in determining entropy of CXCL8-CXCR1 Site-I complex formation. To our knowledge, this is the first study that describes the dynamic characteristics of a class A GPCR N-domain in the ligand-bound state.

## 2. Results

### 2.1. Design of the CXCL8 Monomer and CXCR1 N-Terminal Domain

**Figure 2:**
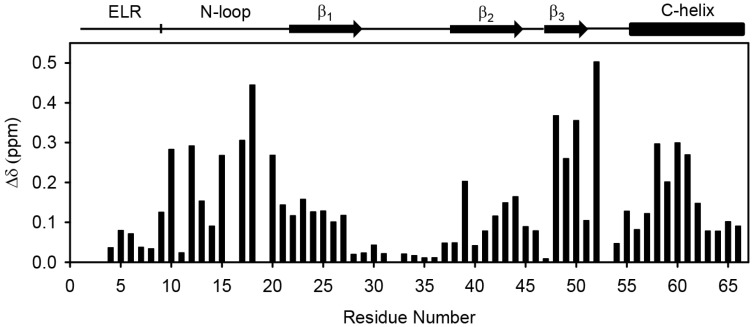
Histogram plot showing CXCR1 N-domain binding-induced chemical shift perturbation in the CXCL8 monomer. Residues 16, 19, 32, and 53 are prolines. The secondary structural elements of the CXCL8 monomer are shown at the top.

Structural characterization of the WT monomer is challenging because the WT exists as a dimer (K_M-D_ ~1 to 10 μM) at >100 μM concentrations used in structural studies. This bottleneck has been tackled by engineering CXCL8 monomers [11,37,38,39,40,41]. In this study, we used the CXCL8 monomer (residues 1–66), which lacks the last six C-terminal residues that stabilize the dimer interface [29,40]. The deleted residues are not involved in receptor binding and are not essential for function [42]. The K_M-D_ of this construct is ~20 mM, and so exists essentially as a monomer (>99%) at the 100 μM concentration used in the relaxation measurements. The solution structure of the 1–66 monomer (PDB ID: 5WDZ) is similar to the full-length monomer that has a methyl group for a backbone amide at the dimer interface [11,12,22] (Figure 1). For the current studies, we used a CXCR1 N-domain peptide corresponding to the first 29 amino acids (29mer) that has been shown to be well behaved and binds the CXCL8 monomer with high affinity [25]. The chemical shift perturbation profile of the CXCL8 (1–66) monomer upon CXCR1 N-domain 29mer binding is shown in Figure 2.

### 2.2. Line Shape Analyses for the ^15^N-CXCL8–CXCR1 N-Domain Interaction

**Figure 3:**
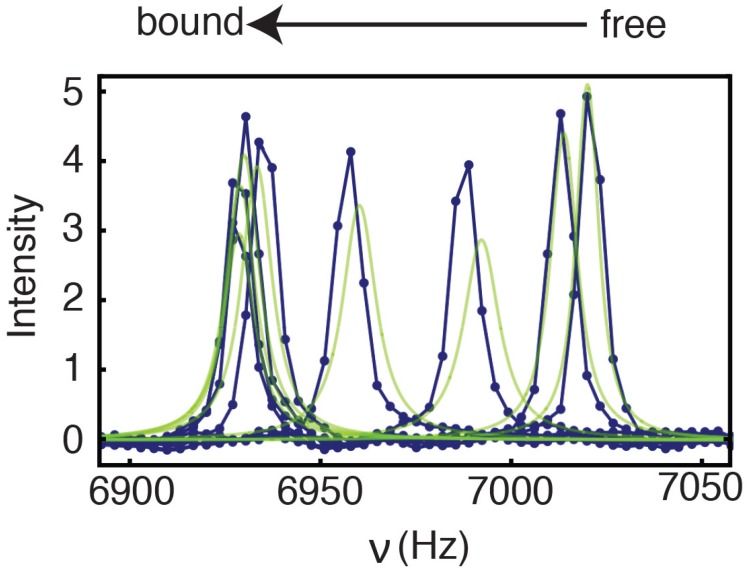
Lineshape analysis for CXCL8 Phe17 ^15^N chemical shift changes from two-dimensional ^1^H-^15^N heteronuclear multiple quantum correlation (HSQC) nuclear magnetic resonance (NMR) spectra upon titration with the CXCR1 N-domain peptide. Experimental data are indicated as blue dots, with the best fits shown as green lines. For the free spectrum, intensity and *R*_2_ value for the F17 resonance were fit to 1.1 × 10^8^ and 20 s^−1^, respectively. These values, the free chemical shift (7020 Hz), and the *K_D_* (5 μM) were fixed for the lineshape analysis. The *k_off_* and bound chemical shifts were numerically optimized during lineshape analysis, giving values of 4319 s^−1^ and 6928 Hz, respectively.

The NMR spectra for residue F17 upon titration of ^15^N-CXCL8 with CXCR1 N-domain are shown in Figure 3. Chemical shift changes for a well-resolved residue F17 yield a *K_D_* value of ~5 μM from the NMR titration, similar to that derived from ITC. For residues C9, F17, and V58, whose ^15^N resonances could be followed through the course of the titration, the *k_off_* values are 2400, 4300, and 6500 s^−1^, respectively, with corresponding *k_on_* values of 4.8, 8.6, and 9.72 × 10^8^ M^−1^ s^−1^. These results indicate that under saturating conditions, the exchange processes for CXCL8 binding of the CXCR1 N-domain occur on a faster time scale (*k_ex_* = *k*_on_[L] + *k_off_*) than those observed in ^15^N relaxation dispersion experiments, as described below.

### 2.3. Relaxation Measurements and Dynamics of the CXCL8 Monomer

The NMR relaxation is sensitive to molecular and internal motions. Therefore, we characterized the backbone dynamics of the CXCL8 monomer and of the CXCR1 N-domain in the free and bound states in the ns–ps to ms–μs time scales by measuring ^15^N-*R*_1_ and ^15^N-*R*_2_ relaxation rates, the heteronuclear {^1^H}^−^^15^N NOE (hetNOE), and ^15^N relaxation dispersion at 600 and 800 MHz field strengths [32,33,34]. The relaxation data were analyzed using the model-independent Lipari–Szabo formalism and its extension that allows describing internal motions at two distinct time scales [43,44,45]. This approach allows for the determination of an order parameter (*S*^2^) that provides a measure of the amplitude of internal motion, overall molecular tumbling of the protein (rotational correlation time, τ_c_), effective correlation times for internal motion on fast (τ_f_) and slow (τ_s_) time scales, and an *R_ex_* parameter that corresponds to the contribution of the ms–μs time scale motions to *R*_2_. Reliable measurements for ms–μs dynamics in the model-free analysis is often challenging under conditions of fast internal motions, and are better quantified using relaxation dispersion experiments [33,34].

The NMR relaxation measurements are typically carried out at high concentrations, as NMR is intrinsically insensitive and requires long acquisition times, and also because reliable quantitation of cross peak intensities collected at longer delays is essential to minimize errors in the relaxation rate measurements. At the same time, measurements must be carried out under conditions at which the backbone resonances are from a single species. This is especially relevant for systems undergoing dimerization, as *R*_2_ is proportional to molecular weight and sensitive to the rate of exchange between the monomer and dimer. *R*_1_ and hetNOE values are sensitive to fast dynamics in the ns–ps time scales but not to motions that occur at slower time scales. Therefore, we obtained *R*_2_ values at 100, 300, and 900 μM concentrations. We observed higher *R*_2_ values at 900 μM compared to 300 μM, and a minimal *R*_2_ difference between 300 and 100 μM, suggesting exchange contributions to *R*_2_ at 900 μM but not at 300 μM or lower concentrations. We detected minor low intensity peaks corresponding to the dimer in the 900 μM spectrum but not in the 300 μM spectrum. Two distinct sets of peaks indicate that the monomer and dimer are in the slow exchange regime in the NMR time scale. The dimer dissociation constant (K_M-D_) was calculated (see Methods section) to be ~20 mM from the heteronuclear multiple quantum correlation (HSQC) peak intensities. Therefore, relaxation measurements of the free CXCL8 monomer were carried out at 100 μM (monomer population >99%) and of the complex at 130 μM. Dimer levels in the bound state are essentially non-existent as the monomer binds the CXCR1 N-domain with much higher affinity.

**Figure 4:**
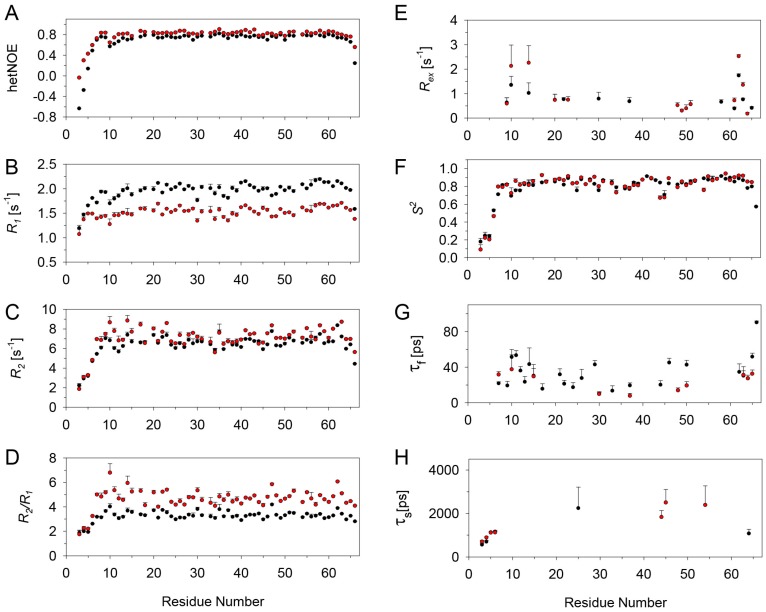
Main chain ^15^N relaxation parameters for the CXCL8 monomer at 600 MHz (black) and 800 MHz (red). (**A**) {^1^H}-^15^N NOE (hetNOE); (**B**) *R*_1_; (**C**) *R*_2_; (**D**) *R*_2_*/R*_1_; (**E**) *R_ex_*; (**F**) *S^2^*; (**G**) τ_f_, and (**H**) τ_s_. The errors in the calculated parameters are plotted as bars.

Relaxation data were obtained for 60 of the 66 backbone amide nitrogens for the CXCL8 monomer in the free state. The missing data include four prolines and the N-terminal residues S1 and A2. The *R*_1_, *R*_2_, *R*_2_/*R*_1_ and hetNOE data at 600 and 800 MHz are shown in Figure 4. The average values of *R*_1_, *R*_2_, and hetNOE at 600 and 800 MHz (in parentheses) are 2.0 s^−1^ (1.5 s^−1^), 6.7 s^−1^ (7.3 s^−1^), and 0.75 (0.82), respectively. The *R*_1_, *R*_2_, and NOE values at both fields are significantly lower than average for the N- and C-terminal regions. Some residues in the loop regions connecting the β-strands also exhibit lower than average values. Elevated *R*_2_/*R*_1_ ratios for I10, S14, R47, and V62 suggest slow ms–μs time scale motions. However, higher *R*_2_/*R*_1_ values can also be the result of anisotropic tumbling of the protein.

Isotropic molecular rotational diffusion is characterized by a single correlation time τ_c_. Axially symmetric diffusion is defined by the ratio of the diffusion rates about the unique and perpendicular axes (D_par_/D_per_), a correlation time τ_c_ ([2D_par_ + 4D_per_]^−1^), and the orientation of the unique axis relative to the coordinates of the molecular structure. Fully anisotropic diffusion is defined by the diffusion rates about three orthogonal axes (D_xx_, D_yy_, and D_zz_) and the orientation of the axis system relative to the structure. The components of the molecular diffusion tensor were estimated from the *R*_2_*/R*_1_ ratio of a select set of 46 residues (described in Methods). For the free CXCL8 monomer, an axially symmetric rotational diffusion model was required to adequately describe the 600 and 800 MHz data. The oblate axially symmetric rotational diffusion model (D_par_/D_per_ = 0.86 ± 0.01), relative to the isotropic model, allowed for a better fit to the 600 MHz data according to the *F*-statistic test (*F* = 3.56, *p* < 0.05). However, the fully anisotropic model relative to an axially symmetric model was not statistically significant (*F* = 1.08, *p* > 0.05). Similar results were obtained for the 800 MHz data, with D_par_/D_per_ = 0.81 ± 0.01.

The relaxation data were fit to five spectral density models for describing the internal dynamics of the CXCL8 monomer with respect to axially symmetric rotational diffusion using the Modelfree and Fast Modelfree programs [46,47]. The model selection strategy uses Monte Carlo numerical simulations to estimate the cumulative probability distributions for statistics characterizing the goodness-of-fit between the dynamical models and the experimental data [46]. The appropriate model for each residue was determined from the quality of the fit. The global rotational correlation time τ_c_ was calculated to be 4.7 ns for both the 600 and 800 MHz data. At 600 MHz, 56 of the 60 residues could be fitted to one of the five models. Residues A35, I40, R47, and W57 did not fit to any model. A total of 21 residues fit to model 1 with an average *S*^2^ of 0.86. Fifteen residues fit to model 2 with an average *S*^2^ of 0.80 and a τ_f_ value ranging from 10 to 90 ps. Four residues fit to model 3 with an average *S*^2^ of 0.87 and *R_ex_* values ranging from 0.4 to 0.8 s^−1^. Nine residues fit to model 4 with an average *S*^2^ of 0.81, *R_ex_* values ranging from 0.4 to 1.8 s^−1^, and τ_f_ values ranging from 10 to 56 ps. Of the thirteen residues that fit to models 3 and 4, only I10, S14, and V62 showed *R_ex_* > 1 s^−1^. The necessity of an *R_ex_* term suggests a conformational exchange process at these sites. Seven residues fit to model 5 with an average *S*^2^ of 0.55 and τ_s_ values ranging from 500 to 3500 ps.

For data collected at 800 MHz, 54 of the 60 residues fit to one of the five motional models. A total of 34 residues fit to models 1 and 2, 13 residues to models 3 and 4, and seven residues to model 5. Some residues with small *R_ex_* values (<0.8 s^−1^) fit to different models between the two fields switching from models 3 and 4 to models 1 and 2 and vice versa, and some that fit to model 5 did not fit any of the models. Residues I10, S14, V62, and E63 showed *R_ex_* >1 s^−1^.

**Figure 5:**
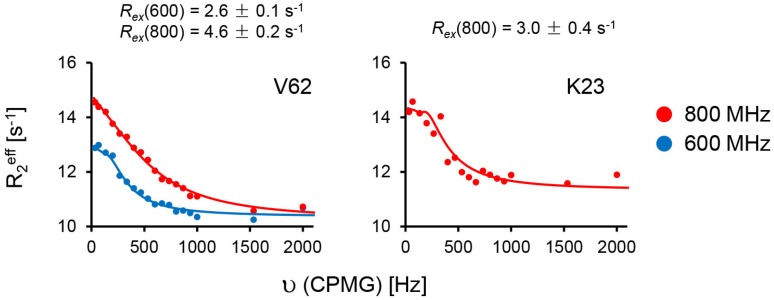
Main chain ^15^N Carr–Purcell–Meiboom–Gill (CPMG) relaxation dispersion curves for free CXCL8 monomer. Plots of effective *R*_2_ relaxation rates (R_2_^eff^) vs. the CPMG frequency (υ_CPMG_) for residues V62 and K23. Solid lines indicate the best fit of the data (solid circles) to a two-site exchange model, with 600 and 800 MHz data shown in blue and red, respectively. The corresponding *R_ex_* values for each of the residues at the two field strengths are also shown.

Several studies have reported that small *R_ex_* terms obtained from fitting the relaxation experimental data to Lipari–Szabo model-free approach must be interpreted with caution, as they may reflect anisotropy of overall tumbling or data fitting artifacts, and are best validated using CPMG relaxation dispersion experiments tailored to detect ms–μs dynamics [48,49,50,51,52,53]. Indeed, CPMG relaxation dispersion data indicate ms–μs dynamics only for V62 and K23 (only at 800 MHz) (Figure 5). Dispersion data for K23 fit to a two-state exchange model with *k_ex_* >> δω, and for V62 to a two-state exchange model with *k_ex_* << δω.

### 2.4. Relaxation Measurements and Dynamics of the CXCL8 Monomer in the Bound State

**Figure 6:**
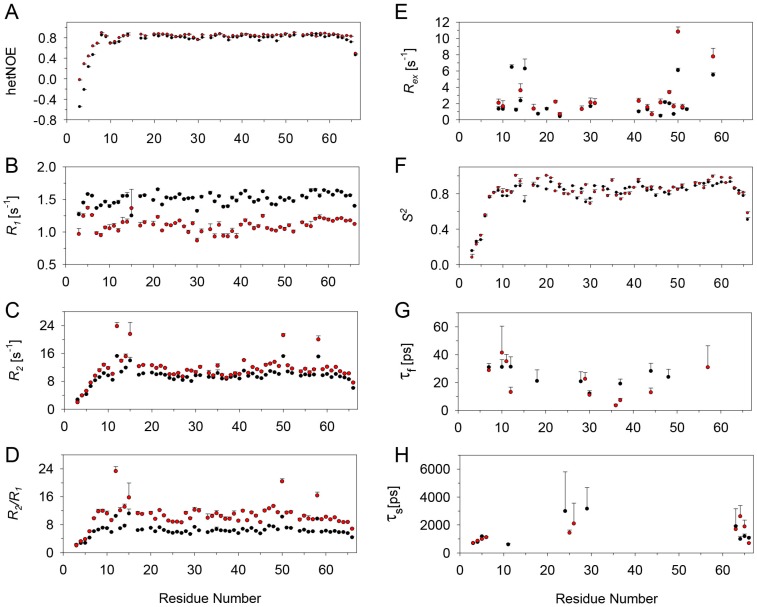
Main chain ^15^N relaxation parameters of bound CXCL8 monomer at 600 MHz (black) and 800 MHz (red). (**A**) {^1^H}^−15^N NOE (hetNOE); (**B**) *R*_1_; (**C**) *R*_2_; (**D**) *R*_2_*/R*_1_; (**E**) *R_ex_*; (**F**) *S^2^*; (**G**) τ_f_, and (**H**) τ_s_. The errors in the calculated parameters are plotted as bars.

Relaxation data could be reliably obtained for 58 of the 66 backbone amide nitrogens in the bound state except for four prolines, S1 and A2, as well as L49 and L51 due to overlap. *R*_1_, *R*_2_, *R*_2_/*R*_1_, and hetNOE data are shown in Figure 6. The average values for *R*_1_, *R*_2_, and NOE at 600 and (800) MHz data are 1.5 s^−1^ (1.1 s^−1^), 9.9 s^−1^ (11.9 s^−1^), and 0.80 (0.83), respectively. Overall trends in the bound state were more or less similar to what was observed in the free state, including small relaxation rates for the termini residues and lower than average values for some of the loop residues. However, high *R*_2_ and *R*_2_/*R*_1_ values were observed for T12, S14, C50, and V58 that were especially apparent in the 800 MHz data.

As the structure of the complex is not available, we fit the relaxation data to the isotropic rotational tumbling model. The overall correlation time τ_c_ for the 600 MHz and 800 MHz was 7.2 ns. Relaxation data for bound CXCL8 were fit to five spectral density models and model selection was carried out as described for the free CXCL8 monomer. For 600 MHz data, 52 of 58 residues fit to one of the five motional models. Residues I22, L25, A35, I40, E55, and V61 could not be fit to any models. Eighteen residues located in the N-loop, 30 s loop, and C-terminal helix fit to model 1 with an average *S*^2^ of 0.88. Four residues fit to model 2 with an average *S*^2^ of 0.81 and a τ_f_ value ranging from 19 to 31 ps. Thirteen residues fit to model 3 with an average *S*^2^ of 0.85 and *R_ex_* values ranging from 0.4 to 6.3 s^−1^. Five residues fit to model 4 with an *S*^2^ of 0.80, and *R_ex_* values ranging from 0.7 to 6.4 s^−1^ and τ_f_ values ranging from 12 to 31 ps. Of the 18 residues that belong to models 3 and 4, fifteen show *R_ex_* values > 1 s^−1^. T12, C50, and V58 show *R_ex_* values > 5 s^−1^. T12 in the N-loop and C50 in the β_3_-strand are proximal to each other in the structure. Residues Y13 and S14 that are adjacent to T12, and V41, R47, E48, and D52 that are clustered around C50 in the β_3_-strand also show ms–μs motions (1 s^−1^ < *R_ex_* < 2.5 s^−1^). C9 and I10 also show *R_ex_* ~1 s^−1^. Given that the signal intensity of K15 is weak in the bound spectrum, the estimated relaxation parameters showed concomitant large errors. However, a large *R*_2_ value of 14 s^−1^ indicates the presence of conformational exchange. Twelve residues fit to model 5, with an average *S*^2^ of 0.60 and τ_s_ values ranging from 594 to 4730 ps. For 800 MHz data, 52 of the 60 residues fit to one of the five motional models. A total of 24 residues fit to models 1 and 2, 17 residues fit to models 3 and 4, and 11 residues fit to model 5. Differences in the selected motional model between the two fields are due to some residues with low *R_ex_* values (<0.6 s^−1^) switching between models 3 and 4 to simpler models and vice versa, and some fitting model 5 did not fit to any of the models.

**Figure 7:**
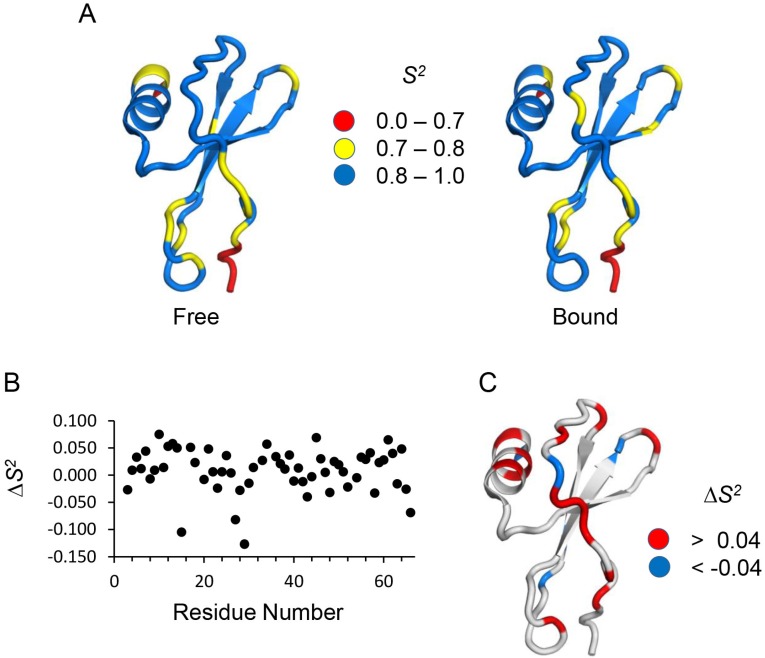
Order parameters for free and bound CXCL8 monomer. (**A**) Molecular plot of CXCL8 monomer highlighting the order parameters (*S^2^*) of backbone amides in the free (left) and CXCR1 N-domain bound (right) states; (**B**) Scatter plot showing the difference in *S^2^* (Δ*S*^2^ = *S*^2^_bound_ − *S*^2^_free_) values between the bound and free states; (**C**) Molecular plot of the CXCL8 monomer with Δ*S*^2^ values mapped on the surface. Color code indicates different ranges of Δ*S*^2^ values.

To better describe the structural basis of fast ns-ps motions, we mapped *S*^2^ values onto the CXCL8 backbone structure (Figure 7A). The profiles for the free and bound states are essentially similar. However, a plot of the differences in *S*^2^ (Δ*S*^2^ = *S*^2^_bound_ − *S*^2^_free_) shows that most residues have higher *S*^2^ in the bound state (Figure 7B,C). Higher Δ*S*^2^ values indicate that the monomer in the bound state is more structured and rigid in the ns–ps time scale.

**Figure 8:**
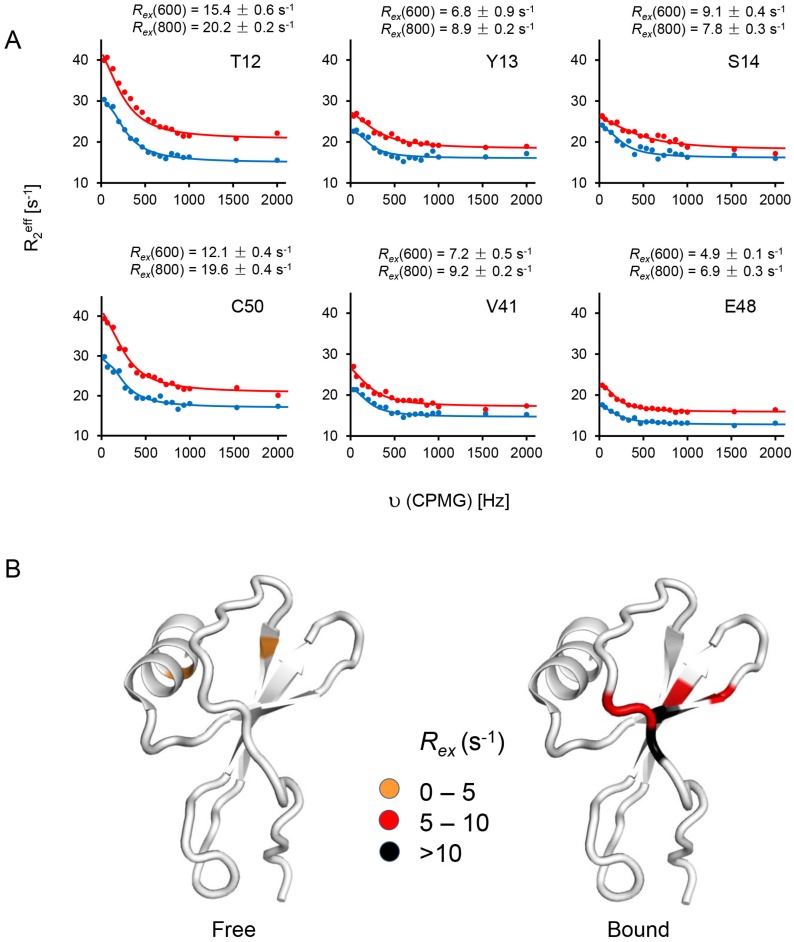
Main chain ^15^N CPMG relaxation dispersion for the CXCL8 monomer bound to CXCR1 peptide. (**A**) Plots of effective *R*_2_ relaxation rates (R_2_^eff^) vs. the CPMG frequency (υ_CPMG_) for residues showing chemical exchange. Solid lines indicate the best fits of the data to a two-site exchange model, with 600 and 800 MHz data shown in blue and red, respectively; (**B**) Structure of the CXCL8 monomer highlighting residues undergoing chemical exchange in the free (left) and bound (right) states. Color code indicates different ranges of *R_ex_* values. The corresponding *R_ex_* values for each of the residues at the two field strengths are also shown.

The CPMG relaxation dispersion experiments indicate the presence of μs–ms timescale dynamics for T12, Y13, S14, V41, E48, and C50. In particular, high *R_ex_* values of ~20 s^−1^ (800 MHz) for T12 and C50 are striking (Figure 8).

All of these residues fit to a two-state exchange model with *k_ex_* >> δω. Dispersion data for K15 and V58 data could not be fit due to weak peak intensities. K15 showed high *R*_2_ and *R*_2_/*R*_1_ values, comparable to those for T12, though the errors in these measurements are larger due to weak peak intensities. In the free state, none of these residues, except S14, showed conformational exchange, indicating that binding is coupled to increased dynamics. Increased ms–μs dynamics in the bound state is unusual, considering dynamics are generally quenched in the bound complexes. These residues are clustered in the structure—whereas T12, Y13, and S14 are part of the binding interface, others are in the proximity of the binding domain. Chemical exchange for a subset of residues in the binding domain suggests a rearrangement of the local structure due to the binding interface becoming more hydrophobic and/or a coupled network that is dynamically primed for conformational selection. For instance, different conformers may reflect how the CXCR1 N-domain-bound monomer is poised to interact with Site-II residues and/or reflect conformational states that preferentially activate G-protein or β-arrestin signaling pathways. The Cys9-Cys50 disulfide links Site-I and Site-II, and it is possible that the disulfide functions as a conformational switch on receptor binding. It is interesting that Cys9 does not exhibit slow dynamics, indicating intrinsic asymmetry of the disulfide. CXCL8 variants that contain the non-natural cysteine analogs penicillamine and homocysteine in either of the disulfides have shown that the individual cysteines of the disulfide play differential roles in function [54]. Chemical exchange for cysteines and residues in the proximity of disulfides has also been observed in other chemokines [55,56,57,58,59].

The relaxation dispersion data can also provide insights into exchange rates (*k_ex_*) and relative amounts of the minor population. For the CXCL8 monomer-CXCR1 N-domain complex, we calculated *k_ex_* to be 900–2000 s^−1^ and a minor population as 1 to 2%. Importantly, while the HSQC spectrum for the CXCL8 monomer shows large ^15^N chemical shift changes for F17 upon binding the CXCR1 N-domain, the ^15^N relaxation dispersion profiles are flat with no contribution from chemical exchange. This indicates that CXCL8 μs–ms timescale internal motions induced upon receptor binding are separate from the exchange process when the receptor-binding site on CXCL8 is fully saturated.

The low *S*^2^ (<0.6) observed for ELR residues both in the free and bound states, indicate that these residues undergo large amplitude motions in the ns–ps time scales. The N-terminal ELR residues in the CXCL8 dimer also show significant dynamics [20], indicating these residues play no direct role in Site-I binding and that the dynamics could be critical for Site-II interactions. Some N-loop and 30s loop residues show low NOEs and/or altered *R*_1_ values indicating a role for fast dynamics in mediating Site-I interactions and that residues remote from the binding site also influence binding.

### 2.5. Relaxation Measurements and Dynamics of the CXCR1 N-Domain

**Figure 9:**
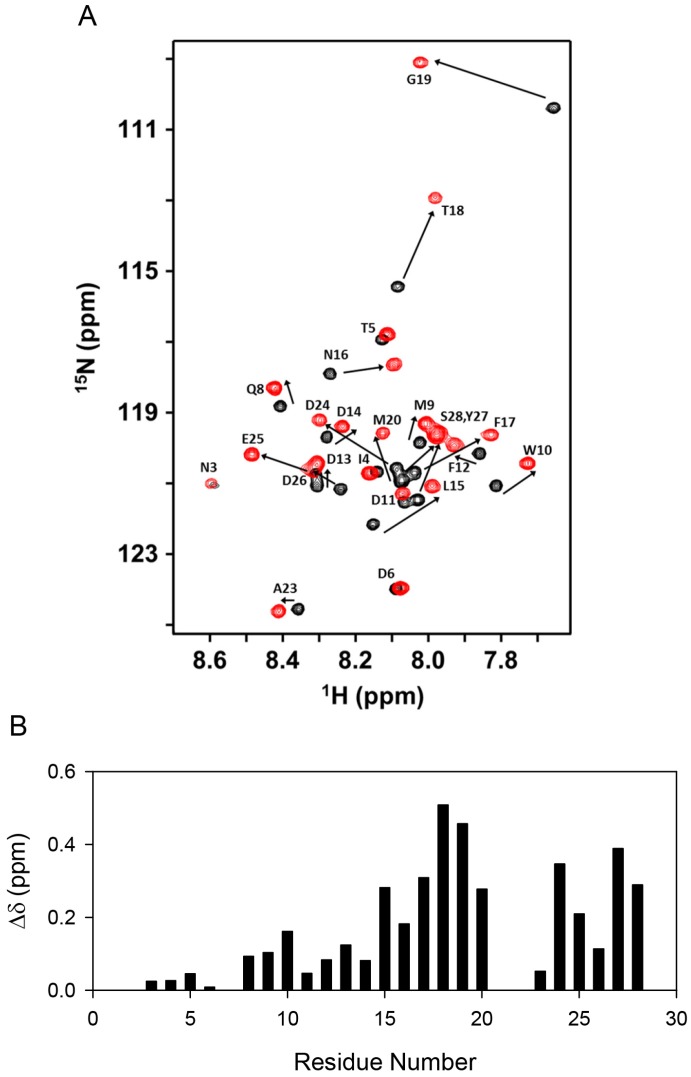
Binding of the CXCL8 monomer to the ^15^N-CXCR1 N-domain. (**A**) ^1^H-^15^N HSQC of the CXCR1 N-domain spectrum showing CXCL8 monomer binding-induced chemical shift change. The unbound peaks are in black and the final bound peaks are in red; (**B**) Histogram plot showing binding-induced chemical shift perturbation in the CXCR1 N-domain. Residues 1 and 2 are not observed.

**Figure 10:**
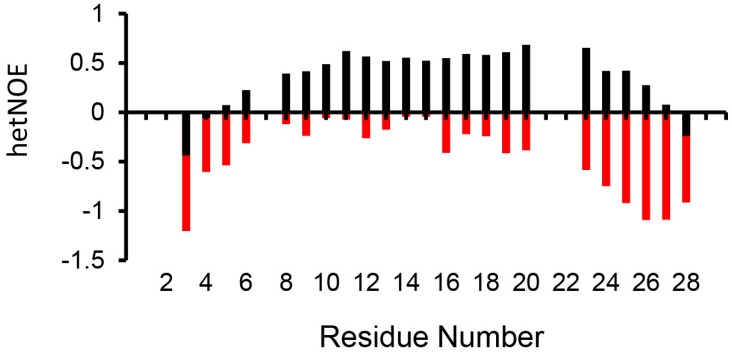
^1^H-^15^N NOE data of the CXCR1 N-domain in the free (red) and CXCL8 monomer-bound (black) states.

The NMR spectra of the CXCR1 N-domain 29mer in the free and bound state show well-dispersed peaks (Figure 9A). A single set of peaks in the bound state indicates that the binding is in the fast exchange regime in the NMR time scale. The chemical shift perturbation profile reveals minimal changes for the first fourteen residues and significant changes for the remaining sequence (Figure 9B). Relaxation data were obtained for a total of 23 residues out of 29 CXCR1 N-domain residues in the free and bound states at 600 MHz. The missing residues include the N-terminal M1 and S2, and four prolines. In the unbound state, negative hetNOEs for all residues indicate that the CXCR1 N-domain is unstructured (Figure 10). Chemical shifts are also characteristic of an unstructured peptide. Previous studies have also shown that the CXCR1 N-domain is unstructured in the free state [15,29].

**Figure 11:**
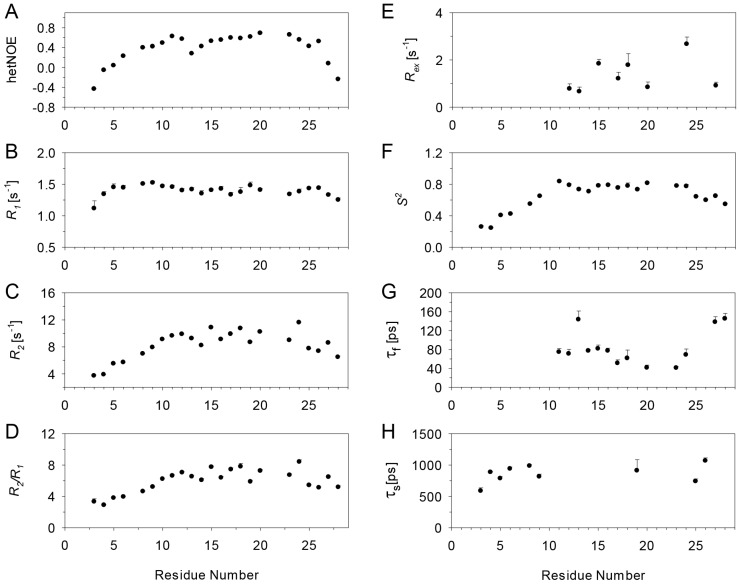
Plot of relaxation parameters for the CXCR1 N-domain bound to the CXCL8 monomer at 600 MHz. (**A**) {^1^H}-^15^N NOE (hetNOE); (**B**) *R*_1_; (**C**) *R*_2_; (**D**) *R*_2_*/R*_1_; (**E**) *R_ex_*; (**F**) *S^2^*; (**G**) τ_f_, and (**H**) τ_s_. The errors in the calculated parameters are plotted as bars.

The relaxation data for the bound state indicate that the CXCR1 N-domain is structured, with distinct differences in dynamic properties at a residue-specific level (Figure 11). Most residues except those in the N-terminus show positive hetNOEs, *R*_1_ values are essentially the same across the sequence except for N- and C-termini, but *R*_2_ values vary and show a complex profile (Figure 11). Calculation of the dynamic parameters indicates that all residues except W10 fit to one of the five models. The average *S*^2^ is 0.65, with residues D11 to D24 showing *S*^2^ of 0.77 that are comparable to those observed in globular proteins. None of the residues fit to models 1 or 3. Five residues (D11, D14, N16, A23, S28) fit to model 2 with an average *S*^2^ of 0.78 (S28 which had a low *S*^2^ of 0.55 was excluded) and τ_f_ values ranging from 40 to 80 ps. Eight residues (F12, D13, L15, F17, T18, M20, D24, and Y27) fit to model 4. These residues had an average *S*^2^ of 0.76, τ_f_ values ranging from 40 to 140 ps, and *R_ex_* values ranging between 1–2 s^−1^ with the exception of D24 that had a larger value of 2.7 s^−1^. Considering that several residues in the proximity of those that fit to model 4 fit to model 2, it is not obvious whether the *R_ex_* values from the fit reflect chemical exchange or are due to fitting artifacts. The CPMG relaxation measurements failed to show any evidence for chemical exchange suggesting the latter. Nine residues (N3 to M9, G19, E25, D26) fit to model 5, and not surprisingly, all residues except G19 in this category are either in the N- or C-termini.

Previous chemical shift perturbation measurement studies using receptor constructs of varying length [25] and our preliminary intermolecular NOE data (data not shown) also indicate that the first nine CXCR1 N-domain residues are not involved in direct binding interactions. In sum, our data provide compelling evidence that the CXCR1 N-domain is structured and undergoing conformational fluctuations on the ns–ps time scales.

### 2.6. Thermodynamics of Binding

**Figure 12:**
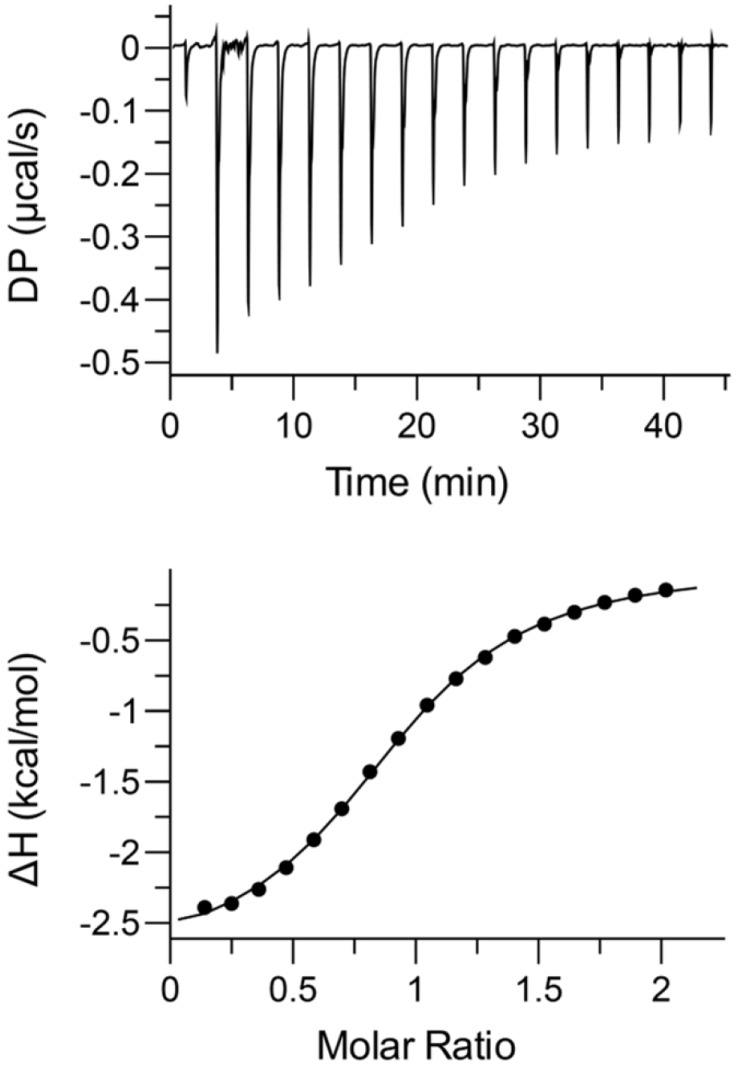
Isothermal titration calorimetry (ITC) titration profiles of CXCR1 N-domain binding to the CXCL8 monomer. Top panel represents the ITC thermogram and the lower panel the fitted binding isotherm. ∆H: enthalpy.

Isothermal titration calorimetry (ITC) measures binding-induced heat changes from which free energy of binding (∆G), enthalpy (∆H), entropy (∆S), and stoichiometry (*n*) can be obtained in a straightforward manner [60]. The binding isotherm of the CXCR1 N-domain binding to the CXCL8 monomer is shown in Figure 12. The data fit best to a single-binding site model, yielding a stoichiometry of 0.93 ± 0.03, *K_D_* = 8.4 ± 0.5 μM, ΔH = −2.6 ± 0.2 kcal/mol, and T∆S = −4.3 ± 0.2 kcal/mol, indicating both enthalpic and entropic factors promote binding. Similar results were observed for binding of the CXCL8 (1–66) monomer to a CXCR1 N-domain 38mer peptide [22]. Dynamic characteristics from NMR measurements provide some insights into the entropy of binding. Whereas higher *S^2^* values in the bound state can be interpreted as entropically disfavored, increased slow dynamics of several residues in the bound state can be interpreted as entropically favored and promote binding.

## 3. Discussion

The GPCRs play diverse roles in all aspects of human physiology, act as conduits between the extracellular and intracellular worlds, and are important drug targets for a wide variety of diseases. Chemokines are unusual agonists for class A GPCRs, as conventional ligands tend to be small molecules such as biogenic amines and those related to taste and smell with a rigid scaffold. Whereas small molecule agonists activate the receptor by binding Site-II, chemokines also bind the receptor N-terminal domain (Site-I). A subset of seven chemokines with a signature N-terminal ‘ELR’ motif function as CXCR1 and CXCR2 ligands. ELR chemokines exist as monomers and dimers, and previous studies have established that the activity of the monomers and dimers vary not only for a given chemokine but also between chemokines and between receptors [4,10,61]. Structure-function studies have shown that Site-I interactions play a fundamental role in determining affinity and specificity. The CXCL8 monomer alone is a potent CXCR1 agonist and differences in monomer vs. dimer affinity have been attributed to Site-I interactions [25,26].

It is well known that GPCRs, including chemokine receptors, are conformationally dynamic and exist as a conformational ensemble [62,63]. In fact, this conformational plasticity has been a major road block in determination of their native structures. Conformational dynamics play a fundamental role in macromolecular recognition and function. In this study, we characterized the dynamics properties of the CXCL8 monomer and CXCR1 residues, with a particular focus on Site-I interactions. Our studies show striking differences in the dynamic characteristics between the free and bound states for both the ligand and the receptor. Our relaxation data indicate that the CXCL8 monomer is more structured on the ns–ps time scale as is evident from increased *S*^2^ in the bound state. At the same time, several residues including those in the N-loop that mediate Site-I interactions showed elevated chemical exchange, indicating that fast and slow dynamics play opposing roles in driving the binding process. The relaxation data indicate the CXCR1 N-domain peptide lacks structure in the free form with central residues (11 to 24) becoming structured in the bound state. Several N-terminal and C-terminal residues remain dynamic showing large amplitude motions in the bound state. These data suggest an entropic penalty in the bound state due to structuring of the central residues within the central domain.

The dynamic fluctuations of proteins are intimately coupled to the thermodynamics of their interactions, bearing in mind that the free energy of binding is a function of the system and includes protein, ligand, solvent, and counter ions. The enthalpy of binding is largely dictated by van der Waals and electrostatic interactions, the latter including hydrogen bonding, ionic, and solvent interactions, whereas the entropy of binding is determined by changes in the mobility of the protein backbone and side chain, as well as rearrangement or release of solvent water molecules and ions [64]. The structure of the CXCR1 N-domain peptide-bound CXCL8 dimer and modeling studies indicate that packing interactions involving aromatic and hydrophobic residues both from the chemokine and the receptor, and polar and H-bonding interactions contribute to enthalpy of binding [31]. Our own, and previously published ITC data, show that both enthalpic and entropic factors promote binding [22]. Interestingly, binding of a rabbit CXCR1 N-domain peptide to the CXCL8 monomer is enthalpically favored but entropically disfavored [29]. Both rabbit and human CXCR1 N-domain peptides bind the CXCL8 monomer with similar affinity, and NMR data suggest that they interact with the same CXCL8 residues [25,29,30]. Their sequences reveal several conserved residues but also show differences, suggesting these differences differentially impact enthalpy and entropy, but not the overall free energy of binding. Besides backbone dynamics, characterization of side chain methyl dynamics in several classes of proteins has also shown that they significantly contribute to conformational entropy and affinity [65]. Whether the side chain dynamics impact CXCR1 N-domain-CXCL8 monomer complex formation is not known. The structure of the CXCR1 N-domain bound CXCL8 monomer is not known, and our current studies are focused towards this goal. Knowledge of reorganization of water and counter ions is harder to come by, and computational simulations such as molecular dynamics (MD) can provide these insights once the structure of the complex becomes available. Collectively, these observations indicate factors that determine dynamics and thermodynamics are quite complex and intimately coupled, and experimentally derived data using NMR and ITC techniques as outlined here can provide some insights towards understanding their roles in binding and function.

Dynamic properties of several chemokines, some in the monomeric state, some in the dimeric state, and some under conditions where both monomers and dimers exist, have been reported [55,56,57,58,59,66,67,68,69]. These studies, in general, show that the Site-I N-loop residues are conformationally dynamic, but also indicate that the dynamic features of each chemokine are quite distinct. For instance, dynamic properties of the three CCR3 chemokine agonists, eotaxin, eotaxin-2, and eotaxin-3, are different indicating that dynamics fine-tunes chemokine-specific binding and functional response [55,56,57,67].

A previous study has reported relaxation properties of a 1 mM CXCL8 L25Y/V27R monomer bound to a CXCR1 N-domain 21mer peptide [21]. This study did not provide residue-specific dynamics of the free or bound chemokine or discuss model fitting for internal motions. The authors report some of the protein exists as a dimer under the experimental conditions, and they observed *R_ex_* for several residues that were attributed to the monomer–dimer equilibrium. There was no discussion of CPMG experiments for CXCL8 in the bound state. David et al. have reported dynamics of the native CCL2 dimer and CCL2 monomer bound to the CCR2 N-terminal sulfopeptide [69]. CCL2 is a CC chemokine and only the monomeric form is competent for receptor activation as dimerization occludes the Site-I receptor-binding site. In the bound state, several CCL2 residues including those in the N-loop and 30s-loop show elevated ms–μs dynamics [69]. These observations suggest that increased slow dynamics could be a shared mechanism between CXC and CC chemokines. However, dynamics characterization of other CXC and CC chemokines is needed to confirm whether this is the case, and these studies will also provide additional insights into how residue-specific similarities and differences fine-tune receptor affinity, selectivity, and specificity.

## 4. Materials and Methods

### 4.1. Protein Expression and Purification

The CXCL8(1–66) monomer and CXCR1 N-domain 29mer peptide were recombinantly expressed in *Escherichia coli* strain BL21(DE3) and purified as discussed earlier [25]. Isotopically labeled proteins were produced by growing *E. coli* cells in minimal medium containing labeled nitrogen and carbon sources. The purity and molecular weight of the proteins and peptides were confirmed using matrix-assisted laser desorption/ionization mass spectrometry (MALDI-MS).

### 4.2. Nuclear Magnetic Resonance Experiments

Protein and peptide samples were prepared in 50 mM sodium phosphate buffer at pH 6.0, containing 1 mM sodium azide and 10% ^2^H_2_O (*v*/*v*). For the NMR experiments of the free proteins, the concentration of the CXCL8 monomer and CXCR1 N-domain were 100 μM and 1 mM, respectively. In the receptor-bound sample, ^15^N-CXCL8 monomer and CXCR1 N-domain concentrations were 130 μM and 1 mM, respectively (1:8 molar ratio). In the chemokine-bound sample, ^15^N-CXCR1 N-domain and unlabeled monomer concentrations were 210 μM and 800 μM, respectively (~1:4 molar ratio). The chemical shifts of the CXCR1 N-domain were determined using standard heteronuclear and triple resonance methods. NMR experiments were recorded at 30 °C on a Bruker 600 MHz (Billerica, MA, USA) equipped with a QCI cryoprobe. The relative amounts of monomeric and dimeric species were determined from the ^1^H-^15^N HSQC peak intensities of a subset of well-dispersed peaks of a CXCL8 monomer sample at 900 μM concentration. The M-D equilibrium was calculated using the following formula [70]:

*K_D_* = [M]^2^/[D] = 2P_t_f^2^_M_/f_D_ = 2P_t_f^2^_M_/(1 − f_M_) (1)

where [M] and [D] are the molar concentrations of the monomer and dimer respectively, P_t_ is the total protein concentration (in monomer molecular mass), and f_M_ and f_D_ are the measured fraction of the monomer and dimer from their peak intensities respectively.

### 4.3. Nuclear Magnetic Resonance Line Shape Analysis

The main chain amide chemical shift changes of ^15^N-CXCL8 upon addition of the CXCR1 N-domain were followed using 2D ^1^H-^15^N HSQC NMR spectra. Titration of 200 μM ^15^N-CXCL8 was conducted through addition of increasing amounts of stock solution containing 2.5 mM unlabeled CXCR1 peptide. Concentrations of protein and peptide for the titration points were 200, 199.2, 196.5, 192.3, 185.2, 175.4, 156.3, and 135.1 μM for CXCL8, and 0, 13.9, 61.9, 134.6, 259.3, 429.8, 765.6, and 1135.1 μM, with corresponding peptide/protein molar ratios of 0, 0.1, 0.3, 0.7, 1.4, 2.5, 4.9, and 8.4. The combined ^15^N and ^1^H chemical shift changes (Δδ) were determined as Δδ = [(Δδ^15^N/5)^2^ + (Δδ^1^H^N^)^2^]^1/2^, where Δδ^15^N and Δδ^1^H are the respective ^15^N and ^1^H chemical shift changes in ppm. Δδ values were fit to a 1:1 binding model to determine the protein-peptide interaction constant (*K_D_*), as previously described [35]. For residues with clearly resolved and intense resonances in the HSQC spectra during the course of the titration (C9, F17, V58), line shape analyses for two-site chemical exchange were conducted as previously described using the Bloch–McConnell equations [36]. Combined with the *K_D_* determined from chemical shift analyses described above, line shape analyses yield the kinetics of binding (*k_on_* = *k_off_*/*K_D_*), through fitting of *k_off_*.

### 4.4. Relaxation Measurements

^15^N-*R*_1_ and -*R*_2_ relaxation rates, hetNOE, and relaxation dispersion experiments for the CXCL8 monomer and CXCR1 N-domain 29mer in the free and bound states were recorded at 25 °C on a Bruker 600 MHz (equipped with a quadruple resonance QCI cryoprobe) and Bruker Avance III 800 MHz (equipped with a triple resonance TXI cryoprobe) spectrometers. All relaxation data were collected as single scanned interleaved pseudo three-dimensional (3D) experiments. The spectra were processed using NMRPipe and analyzed using NMRView. Steady state heteronuclear NOE values were obtained by recording spectra with and without proton saturation. For the free and bound CXCL8 monomer, and the bound CXCR1 N-domain, a saturation time of 3 s and delay of 3 s between scans were used. In the case of the free CXCR1 N-domain, a saturation time of 5 s and a delay of 10 s were used.^15^N-*R*_1_ and -*R*_2_ data were acquired using 10 different relaxation delays (*T*_1_: 10, 50, 100, 150, 200, 250, 300, 400, 600, and 800 ms; *T*_2_: 16.1, 32.1, 48.2, 64.3, 80.3, 96.4, 112.5, 128.5, 160.6, and 192.8 ms). The hetNOE values were calculated using the hetNOE analysis routine in NMRView. ^15^N-*T*_1_ and *T*_2_ relaxation times were calculated from non-linear least-square fits of the changes in cross peak intensities to a two-parameter exponential decay using a suite of Mathematica notebooks [71].

Rotational correlation times (τ_c_) were initially calculated on a per residue basis by minimizing the differences between the experimental and the calculated *T*_1_, *T*_2_, and hetNOE values using the spectral density function. The global rotational correlation times (τ_c_) are the averages from per residue fits excluding residues with NOE < 0.65. The components of the molecular rotational diffusion tensors were estimated from the *R*_2_/*R*_1_ ratios of individual residues for the case of isotropic and axially symmetric diffusion tensors using the programs r2r1_diffusion after excluding residues with hetNOE < 0.65 and those with short *T*_2_ values and potentially undergoing conformational exchange [46,51]. The program quadric_diffusion was used for the case of fully anisotropic diffusion tensor. The diffusion model that adequately represents the relaxation data was determined using an *F*-statistic and the statistical significance of the models were determined by a *p*-value calculation [72]. The relaxation data were analyzed using Lipari–Szabo formalism, and fit to different models using the Fastmodelfree and Modelfree suite of programs [46,47].

CPMG relaxation dispersion profiles were obtained by recording spectra with varying CPMG pulse frequencies (υCPMG 0, 33.3, 66.6, 133.3 × 2, 200.0, 266.6, 333.3, 400.0, 466.6, 533.3 × 2, 600.0, 666.6, 733.3, 800.0, 866.6, 933.3 × 2, 1000.0, and 2000.0 Hz) using a 60 ms constant CPMG relaxation delay (T_CPMG_). The relaxation dispersion data were fit to two-site chemical exchange with the parameters *k_ex_* (rate of exchange), δω (chemical shift difference between exchanging states), and the populations of the individual states (*p*_A_, *p*_B_). Peak intensities extracted from the individual spectra were analyzed using the NESSY program [73]. The data were fit to two different two-site exchange models, the fast-limit with *k_ex_* >> δω, and the slow-limit with *k_ex_* << δω. Model selection was performed using AICc (Akaike’s information criteria with second order correction).

### 4.5. Isothermal Titration Calorimetry

Thermodynamic parameters of CXCR1 N-domain binding to the CXCL8 monomer were determined at 25 °C using a Malvern PEAQ-ITC microcalorimeter (Westborough, MA, USA). Titrations were performed by injecting 1 × 0.5 µL and 19 × 2 µL aliquots of 890 μM CXCR1 N-domain peptide to 80 μM CXCL8 monomer prepared in 50 mM sodium phosphate buffer, pH 6.0. The experiments were repeated twice. The raw data were corrected using a buffer control wherein CXCR1 N-domain was titrated into the buffer. The data were analyzed using the software supplied by the manufacturer.
